# Kisspeptin regulates the proliferation and apoptosis of ovary granulosa cells in polycystic ovary syndrome by modulating the PI3K/AKT/ERK signalling pathway

**DOI:** 10.1186/s12905-022-02154-6

**Published:** 2023-01-11

**Authors:** Pingping Sun, Yuemin Zhang, Lilan Sun, Na Sun, Jinguang Wang, Huagang Ma

**Affiliations:** Reproductive Medicine Center, Weifang People’s Hospital, No. 151, Guangwen Street, Kuiwen District, Weifang, 261000 Shandong China

**Keywords:** Kisspeptin, Polycystic ovary syndrome, Granulosa cell, Oxidative stress, Apoptosis, PI3K/AKT/ERK signalling

## Abstract

**Background:**

The development of polycystic ovary syndrome (PCOS) is closely correlated with apoptosis and oxidative stress in ovarian granulosa cells. Kisspeptin plays an important role in reproductive organ function. This study aimed to explore the role of kisspeptin in PCOS and oxidative stress-triggered apoptosis of ovarian granular cells.

**Methods:**

A PCOS rat model was established by injecting dehydroepiandrosterone (DHEA) and feeding the rats a high-fat diet. The RNA and protein levels of kisspeptin were analysed by quantitative PCR, western blotting, and histological staining. Tissue damage was evaluated using haematoxylin and eosin (H&E) staining. The viability and proliferation of human granulosa cell KGN were measured using the cell counting kit-8 (CCK-8) and 5-ethynyl-2′-deoxyuridine (EdU) assays. Cell cycle and apoptosis were analysed by flow cytometry. Oxidative stress was analysed by measuring reactive oxygen species (ROS), malondialdehyde (MDA), glutathione (GSH), superoxide dismutase (SOD), and catalase (CAT) levels.

**Results:**

Kisspeptin was downregulated in the ovarian granulosa cells of PCOS rats compared to those of control rats. Kisspeptin overexpression enhanced KGN cell proliferation and inhibited apoptosis. ROS generation was suppressed by kisspeptin, along with decreased levels of MDA and increased levels of the antioxidants GSH, SOD, and CAT. Kisspeptin activates PI3K/AKT and ERK signalling, and inactivation of ERK1/2 suppresses the protective role of kisspeptin in ovarian granulosa cells.

**Conclusion:**

Kisspeptin improves proliferation and alleviates apoptosis and oxidative stress in ovarian granulosa cells by activating PI3K/AKT and ERK signalling.

## Highlights

1. Kisspeptin expression is decreased in ovaries of PCOS rat
2. Kisspeptin promotes proliferation and inhibits apoptosis of granulosa cells
3. Kisspeptin alleviates oxidative stress of granulosa cells
4. Kisspeptin activates PI3K/AKT/ERK signalling pathway

## Background

Polycystic ovary syndrome (PCOS) is a common endocrine disorder among females of reproductive age [1]. PCOS is characterised by ovulatory dysfunction, polycystic ovaries, and hyperandrogenism, which causes anovulatory infertility and compensatory hyperinsulinaemia [2]. Patients with PCOS often show increased male hormone secretion and elevated inflammation levels [2]. However, the specific pathogenesis and mechanisms of PCOS remain largely unknown. Various studies have revealed that PCOS is accompanied by the accumulation of reactive oxygen species (ROS), suppressed antioxidant function, and elevated oxidative stress, indicating the critical role of oxidative stress in the pathophysiology of PCOS [3, 4]. Moreover, decreased superoxide dismutase (SOD) activity has been observed in follicular fluid and serum collected from patients with PCOS [5].

ROS are produced during multiple physiological processes, as well as during an imbalance between pro-oxidants and antioxidants [6]. Antioxidants such as glutathione (GSH), SOD, and catalase (CAT) maintain ROS at low levels to ensure normal cellular function [6]. Moreover, excessive ROS accumulation can lead to mitochondria-related apoptosis [7–9]. Studies have demonstrated that oxidative stress-induced apoptosis occurs in ovarian granulosa cells in PCOS [10, 11]. Apoptosis and dysfunction of ovarian granulosa cells have been reported to play important roles in PCOS development [12, 13]. Therefore, targeting apoptosis and oxidative stress in ovarian granulosa cells is a plausible method for PCOS treatment.

Kisspeptins are neuropeptides initially identified as tumour suppressors that inhibit tumour cell metastasis [14]. Recent studies have revealed their critical role in regulating the mammalian reproductive system, including puberty onset, gonadotropin secretion, brain sex differentiation, and ovulation by controlling the production of gonadotropin-releasing hormone (GnRH) [15, 16]. However, whether kisspeptin modulates oxidative stress-induced apoptosis of ovarian granulosa cells during PCOS progression remains unclear.

This study investigated the relationship between kisspeptin expression and PCOS using an in vivo rat model and determined the role of kisspeptin in modulating proliferation, apoptosis, and oxidative stress in ovarian granulosa cells. Our study provides new evidences of kisspeptin function during PCOS and presents it as a promising treatment for PCOS.

## Methods

### PCOS rat model

Three-week-old female Sprague–Dawley rats were procured from Jinan Pengyue Experimental Animal Breeding Co., Ltd. (Jinan, China) and maintained under standard housing conditions. Rats were randomly divided into two groups, a dehydroepiandrosterone (DHEA) group and an oil group, with five rats in each group. Rats were subcutaneously injected with DHEA (60 mg/kg in 0.2 ml sesame oil) daily to induce PCOS and fed a 60% (by calories) fat diet [17]. Rats in the oil group were injected with the same volume of sesame oil as the control. After 20 days of treatment, the weight and nose-to-anus length of each rat were recorded. BMI was calculated as body weight (g) divided by the square of nose-to-anus length (cm). Subsequently, the rats were sacrificed, and ovary tissues were collected for subsequent experiments. Animal experiments were conducted in accordance with the guidelines of the Experimental Animal Ethics Committee of our hospital.

### Haematoxylin and eosin (H&E) staining

The collected ovaries were washed twice with phosphate buffer saline (PBS), fixed with 4% paraformaldehyde (Beyotime, China), embedded in paraffin, and cut into 5-μm slices. Tissue samples were stained with haematoxylin and eosin (Beyotime, China). Images were captured using a microscope (Olympus, Tokyo, Japan).

### Immunohistochemical analysis

To examine the expression of kisspeptin in ovary tissues, paraffin-embedded tissues were heated at 98 °C for antigen retrieval, blocked with goat serum, and incubated with anti-kisspeptin antibody (1:100; Abcam, USA) overnight at 4 °C. The protein bands were incubated with biotin-labelled secondary antibodies and visualised using DAB reagent (Beyotime, China). Images were captured using a microscope (Olympus).

### Cell culture

The human ovarian granulosa cell line, KGN, was purchased from Procell (Wuhan, China). Cells were cultured in DMEM/F12 medium (Hyclone, USA) containing 10% FBS (Hyclone) and 1% penicillin/streptomycin at 37 °C in an incubator filled with 5% CO_2_.

### Cell transfection and treatment

Kisspeptin overexpressing vector (KISS-1), shRNA targeting kisspeptin (shRNA-1 and shRNA-2), and scrambled shRNA control were obtained from GenePharma. Ltd. (Shanghai, China). KGN cells were plated in 6-well plates and transfected with the indicated vectors or shRNA using Lipofectamine 3000 (Invitrogen, USA). To inhibit the ERK signalling pathway, KGN cells were treated with the ERK1/2 antagonist PD98059 (5 μM; Selleck, USA) for 24 h.

### Cell counting kit 8 (CCK-8) assay

Transfected cells were placed in a 96-well plate at 5000 cells per well. After incubation for 0, 24, 48, and 72 h, 20 μL CCK-8 reagent (Beytime, China) was added to each well and incubated for additional 2 h, followed by an examination of the optical density at 450 nm using a microplate detector (Bio-Rad, USA).

### 5-ethynyl-2′-deoxyuridine (EdU) staining

For the EdU assay, cells were fixed with 4% paraformaldehyde, permeabilised with 0.5% Triton X-100, and stained with EdU (50 µM) for 3 h. Nuclei were stained with 4′,6-diamidino-2-phenylindole (DAPI; Sigma, USA) for 10 min at room temperature. EdU-positive staining was observed and imaged using a fluorescence microscope (Carl Zeiss, Germany).

### Flow cytometry

Apoptosis and the cell cycle were measured using flow cytometry. For apoptosis, cells were collected and suspended in a binding buffer and labelled with 5 μL Annexin V-FITC (Sigma, USA) and 5 μL propidium iodide (PI; Sigma, USA) for 20 min in the dark. Subsequently, samples were examined using a flow cytometer (BD Biosciences, USA).

For cell cycle detection, cells were fixed with 70% ethanol at 4 ℃ for 3 days, suspended in PBS containing 100 μg/mL PI, 1% Triton-X100, and 100 μg/mL RNase A, stained for 30 min, and detected using a flow cytometer (BD Biosciences, USA).

### ROS generation

Intracellular ROS accumulation was measured via 2′,7′-diclorodihydrofluorescein di-acetate (DCFDA) staining. Briefly, KGN cells were incubated with 10 μM DCFDA in PBS for 25 min at 37 °C, and nuclei were stained with DAPI for 10 min. The cells were collected and analysed using a flow cytometer (BD Biosciences, USA).

### Detection of oxidative stress

To determine oxidative stress, MDA, GSH, SOD, and CAT levels were analysed using commercial kits (Beyotime, China) following the manufacturer’s protocols.

### qRT-PCR assay

Ovary tissues and KGN cells were homogenised using TRIzol reagent (Sigma, USA). A total of 1 µg of RNA was reverse-transcribed to cDNA using High-Capacity cDNA reverse transcription kits (Thermo, USA) according to the manufacturer’s instructions. KISS-1 gene levels were quantified by qRT-PCR using SYBR Green/Mix (Thermo Fisher Scientific, USA) following the 2^−△△Ct^ method. GAPDH was used as an internal control. The primer sequences are listed as follows. Rattus norvegicus KISS-1, 3’- CAGTGTGCTCCAACTACCCA -5′ (forward), 3′-GTGTCCAGAGGCTTGGCTG -5′ (reverse); Rattus norvegicus GAPDH, 3′-CTCTCTGCTCCTCCCTGTTC -5′ (forward), 3′-CGATACGGCCAAATCCGTTC -5’ (reverse); Homo sapiens KISS-1, 3′-GCACTTCTAGGACCTGCCTC-5′ (forward), 3′-GATTCTAGCTGCTGGCCTGTG-5′ (reverse); Homo sapiens GAPDH, 3′-GAATGGGCAGCCGTTAGGAA -5′ (forward), 3′-GAGGGATCTCGCTCCTGGAA -5′ (reverse).

### Western blot

Tissues and cells were lysed with radioimmunoprecipitation assay (RIPA) lysis buffer (SolarBio, China) containing a protease inhibitor cocktail (Invitrogen, USA). Proteins (60 μg) were separated through 8–10% sodium dodecyl sulphate–polyacrylamide gel electrophoresis and transferred onto polyvinylidene fluoride membranes (Millipore, USA). After blocking with 5% non-fat milk, the blots were incubated with primary antibodies against kisspeptin, PI3K, p-PI3K, Akt, p-Akt^Ser473^, ERK1/2, p-ERK1/2, Bax, Bcl-2, cleaved caspase-3, and GAPDH (1:1000; Abcam, USA). Subsequently, the protein bands were incubated with HRP-conjugated secondary anti-rabbit antibody or anti-mouse antibody (1:2000; Abcam, USA) and visualised after incubation with enhanced chemiluminescent reagent (Millipore, USA).

### Statistical analysis

All data are presented as mean ± standard deviation. Comparisons between two or multiple comparisons were performed using Student’s t-test or one-way analysis of variance using SPSS software (version 19.0). Results were considered statistically significant at *P* < 0.05.

## Results

### Kisspeptin is downregulated in ovarian granulosa cells of PCOS rats

**Fig. 1:**
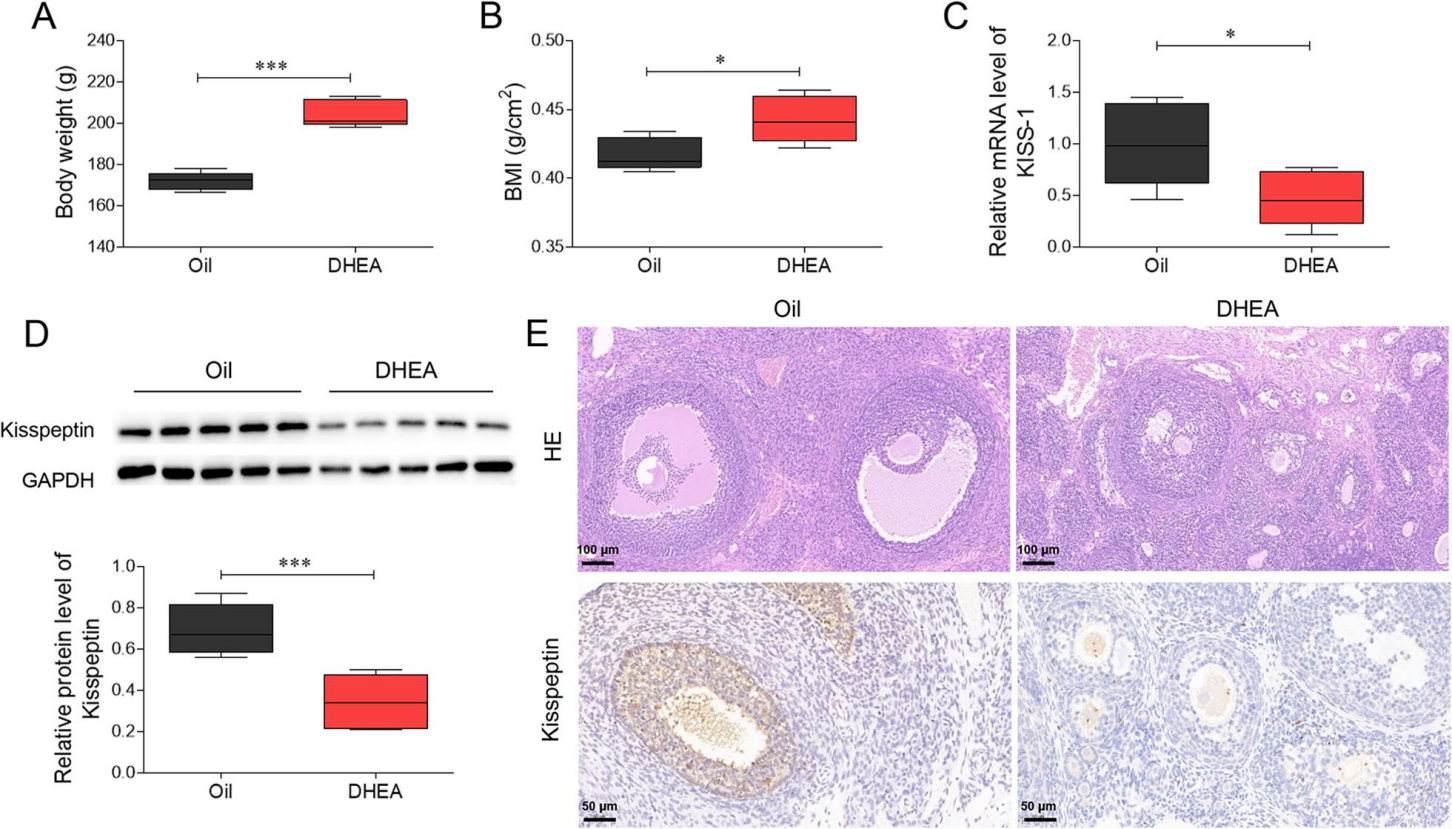
Kisspeptin is downregulated in ovarian granulosa cells of PCOS rats. PCOS rat model was established using DHEA and high-fat diet, oil-fed rats were set as control. **A** The body weight and **B** BMI of rats. **C** The qRT-PCR assay to measure KISS RNA level in ovaries. **D** The western blot to check kisspeptin protein level in ovaries. **E** Histological assessment of tissue damage (H&E staining) and kisspeptin level. *P < 0.05, ***P < 0.001 vs. oil group

We established a DHEA-induced PCOS rat model with oil-treated rats as a control. DHEA rats were fed a high-fat diet to exaggerate PCOS phenotypes. As shown in Fig. 1A, B, body weight and BMI were significantly higher in DHEA rats than in rats in the oil group (*P* < 0.05). Additionally, the RNA and protein levels of kisspeptin were significantly decreased in the ovarian granular cells of PCOS rats compared with those in the oil group (*P* < 0.05, Fig. 1C, D). H&E staining showed considerable tissue damage in the PCOS group, as evidenced by the increased number of immature follicles. Immunohistochemical analysis showed a remarkable downregulation of kisspeptin in the PCOS group compared with that in the oil group (Fig. 1E). These results indicate that decreased kisspeptin expression is correlated with ovarian damage in PCOS rats.

### Kisspeptin promotes the proliferation of ovarian granulosa cell line KGN

**Fig. 2:**
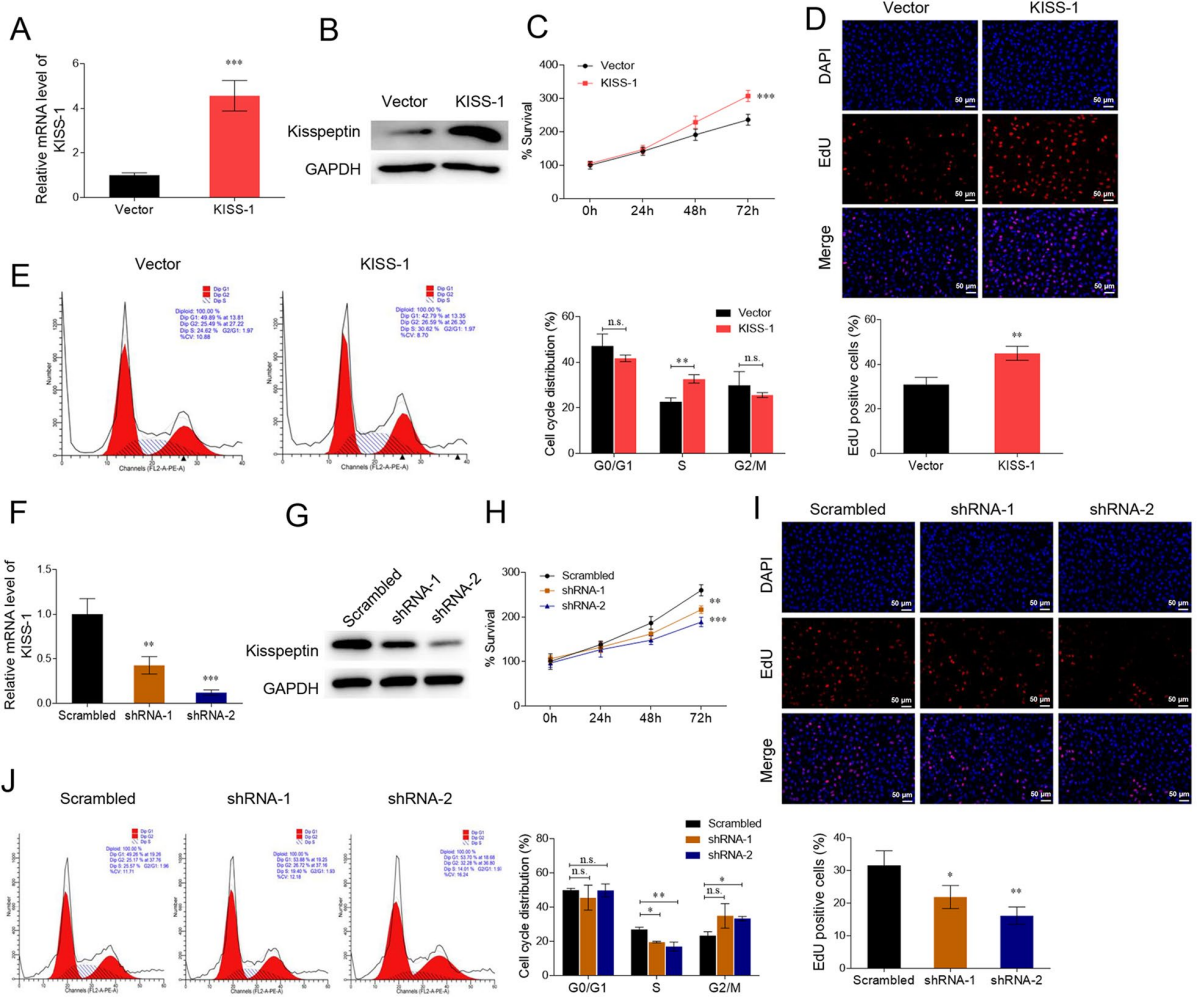
Kisspeptin promotes the proliferation of ovarian granulosa cell line KGN. **A**–**E** KGN cells were transfected with KISS-1-overexpressing vectors or the empty vector. The RNA (**A**) and protein (**B**) levels of kisspeptin were measured. Cell proliferation and cell cycle progression was assessed using CCK-8 (**C**), EdU (**D**), and flow cytometry (**E**). **F**–**J** KGN cells were transfected with KISS-1 shRNAs or the scrambled shRNA. The RNA (**F**) and protein (**G**) levels of kisspeptin were measured. Cell proliferation and cell cycle progression were assessed with CCK-8 (**H**), EdU (**I**), and flow cytometry (**J**). n.s, not significant; *P < 0.05, **P < 0.01, ***P < 0.001

The KGN cell line is a human granulosa-like tumour cell line whose physiological characteristics most closely resemble those of normal ovarian granulosa cells and is frequently used in PCOS studies [18]. To determine the detailed function of kisspeptin in PCOS progression, we examined exogenous kisspeptin expression in the ovarian granulosa cell line, KGN. Transfection with KISS-1 overexpressing vectors significantly increased the RNA and protein levels of kisspeptin in KGN cells (*P* < 0.05, Fig. 2A, B). CCK-8 and EdU staining results showed that, compared with the control cells, kisspeptin overexpression significantly enhanced the viability and proliferation of KGN cells (*P* < 0.05, Fig. 2C, D). Furthermore, kisspeptin overexpression significantly increased the proportion of cells in S phase, indicating enhanced cell proliferation (*P* < 0.05, Fig. 2E). Moreover, we transfected KGN cells with kisspeptin-specific shRNAs to effectively downregulate the RNA and protein levels of kisspeptin (*P* < 0.05, Fig. 2F, G). In contrast to the scrambled control group, kisspeptin shRNAs significantly suppressed the viability, proliferation, and proportion of S-phase KGN cells (*P* < 0.05, Fig. 2H–J).

### Kisspeptin inhibits the apoptosis of ovarian granulosa cell line KGN

**Fig. 3:**
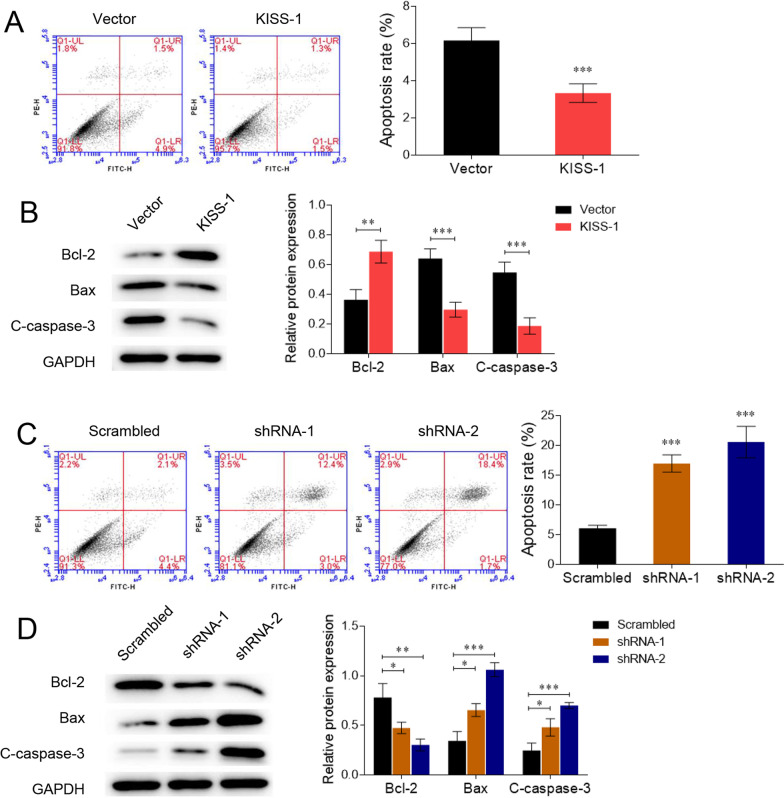
Kisspeptin inhibits the apoptosis of ovarian granulosa cell line KGN. **A**, **B** KGN cells were transfected with KISS-1-overexpressing vectors or the empty vector. Cell apoptosis (**A**) and protein levels of Bcl-2, Bax, and cleaved-caspase 3 (C-caspase-3) (**B**) were detected by flow cytometry and western blotting. **C**, **D** KGN cells were transfected with KISS-1 shRNAs or the scrambled shRNA. Cell apoptosis (**C**) and protein levels of Bcl-2, Bax, and cleaved-caspase 3 (C-caspase-3) **D** were detected by flow cytometry and western blotting. *P < 0.05, **P < 0.01, ***P < 0.001

We examined the apoptosis of KGN cells following transfection with the kisspeptin overexpression plasmid and shRNAs. As shown in Fig. 3A, B, kisspeptin overexpression led to decreased cell apoptosis, upregulation of the anti-apoptotic factor Bcl-2, and downregulation of the pro-apoptotic factors cleaved caspase-3 and Bax (*P* < 0.05). In contrast, kisspeptin knockdown promoted apoptosis, increased cleaved caspase-3 and Bax expression, and suppressed Bcl-2 expression (*P* < 0.05, Fig. 3C, D).

### Kisspeptin inhibits oxidative stress of ovarian granulosa cell line KGN

**Fig. 4:**
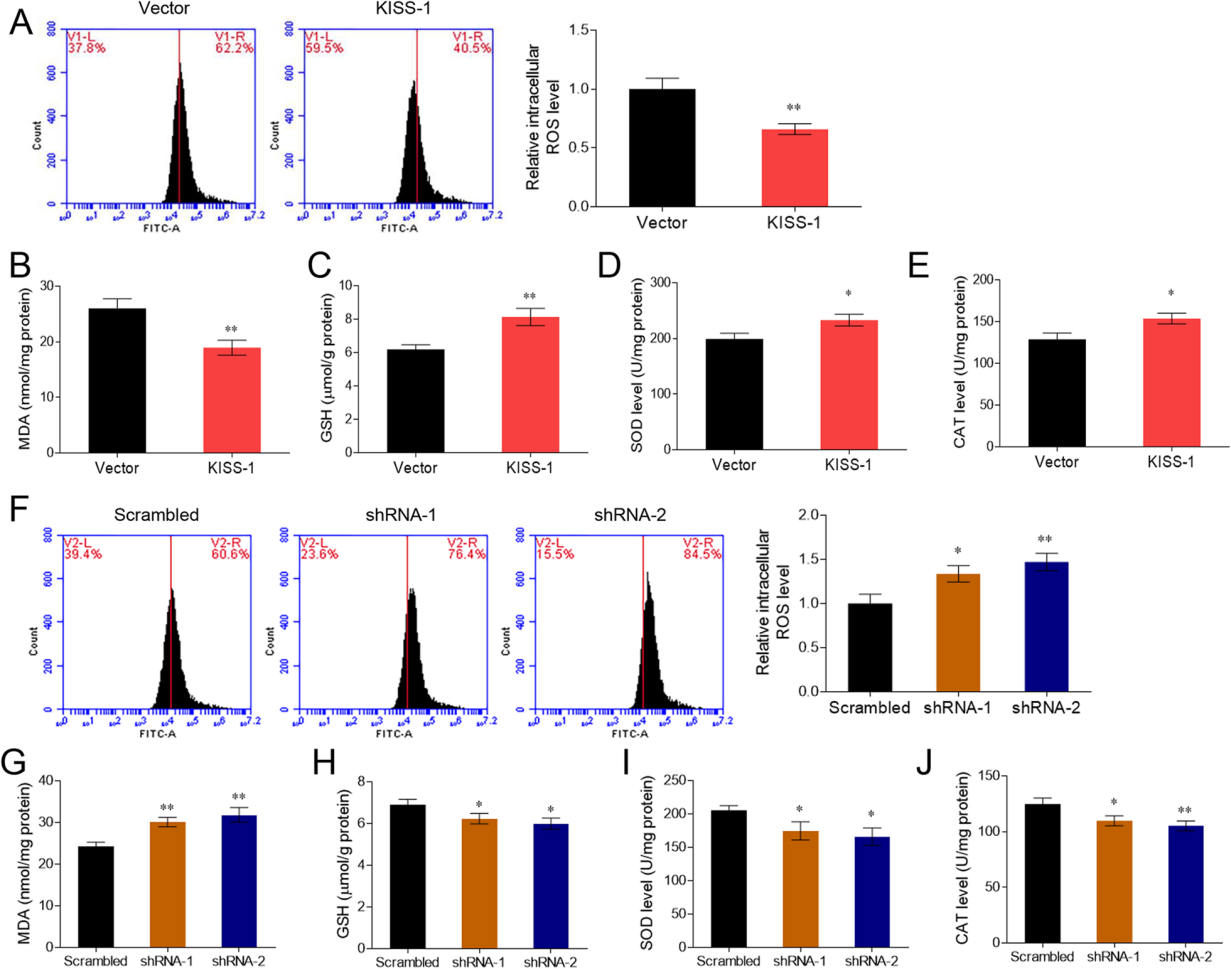
Kisspeptin inhibits oxidative stress of ovarian granulosa cell line KGN. **A**–**E** KGN cells were transfected with KISS-1-overexpressing vectors or the empty vector. The intracellular levels of ROS (**A**), MDA (**B**), and antioxidant enzymes GSH (**C**), SOD (**D**), and CAT (**E**) were measured. **F**–**J** KGN cells were transfected with KISS-1 shRNAs or the scrambled shRNA. The intracellular levels of ROS (F), MDA (**G**), and antioxidant enzymes GSH (**H**), SOD (**I**), and CAT (**J**) were measured. *P < 0.05, **P < 0.01

We determined the effects of kisspeptin on intracellular oxidative stress in the ovarian granulosa cell line KGN. The flow cytometry results demonstrated that kisspeptin inhibited ROS accumulation (*P* < 0.05, Fig. 4A). MDA production was suppressed by kisspeptin overexpression (*P* < 0.05, Fig. 4B), whereas the levels of the antioxidant enzymes GSH, SOD, and CAT were elevated (*P* < 0.05, Fig. 4C–E), suggesting the anti-oxidative effects of kisspeptin. In contrast, kisspeptin knockdown stimulated the accumulation of intracellular ROS and MDA (*P* < 0.05, Fig. 4F, G) and decreased the levels of antioxidant enzymes (*P* < 0.05, Fig. 4H–J).

### Kisspeptin induces the activation of PI3K/AKT/ERK signalling pathway

**Fig. 5:**
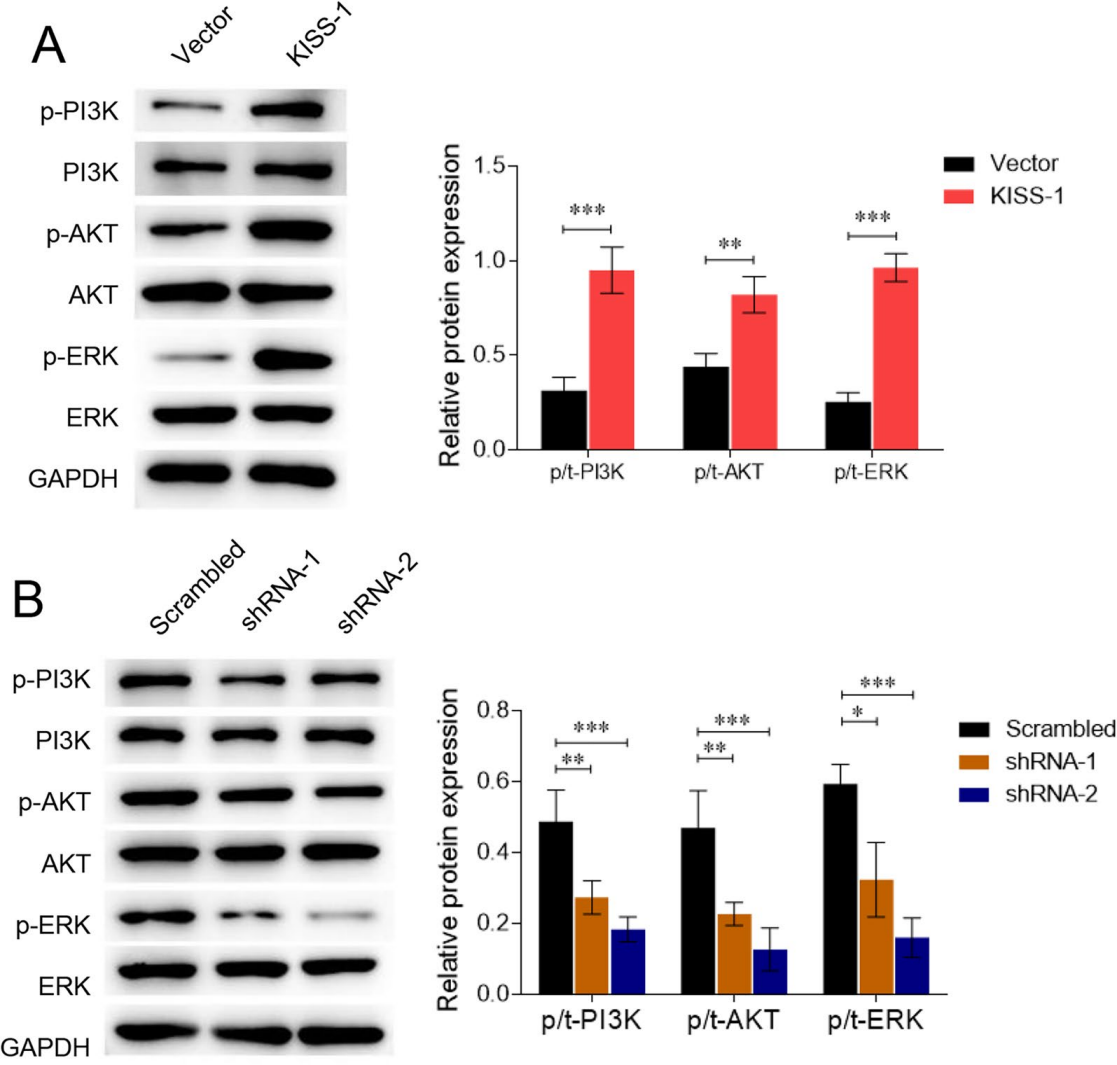
Kisspeptin induces the activation of PI3K/AKT/ERK signalling pathway. KGN cells were transfected with KISS-1-overexpressing vectors (**A**), shRNAs targeting KISS-1 (**B**), or the negative controls. The phosphorylated and total levels of PI3K, AKT, and ERK were measured by western blotting. *P < 0.05, **P < 0.01, ***P < 0.001

Studies have reported the regulatory effect of kisspeptin on PI3K/AKT and ERK signalling pathways [19, 20]. Here, we further analysed whether this regulation exists in KGN cells. We observed that the levels of phosphorylated PI3K, AKT, and ERK in KGN cells were increased in kisspeptin-overexpressing cells, whereas the levels of total PI3K, AKT, and ERK remained unchanged (*P* < 0.05, Fig. 5A), indicating the activation of PI3K/AKT and ERK signalling. In contrast, knockdown of kisspeptin using shRNAs suppressed the levels of p-PI3K, p-AKT, and p-ERK (*P* < 0.05, Fig. 5B), suggesting inactivation of the PI3K/AKT and ERK pathways.

### Kisspeptin promotes KGN cell survival via activation of PI3K/AKT/ERK signalling

**Fig. 6:**
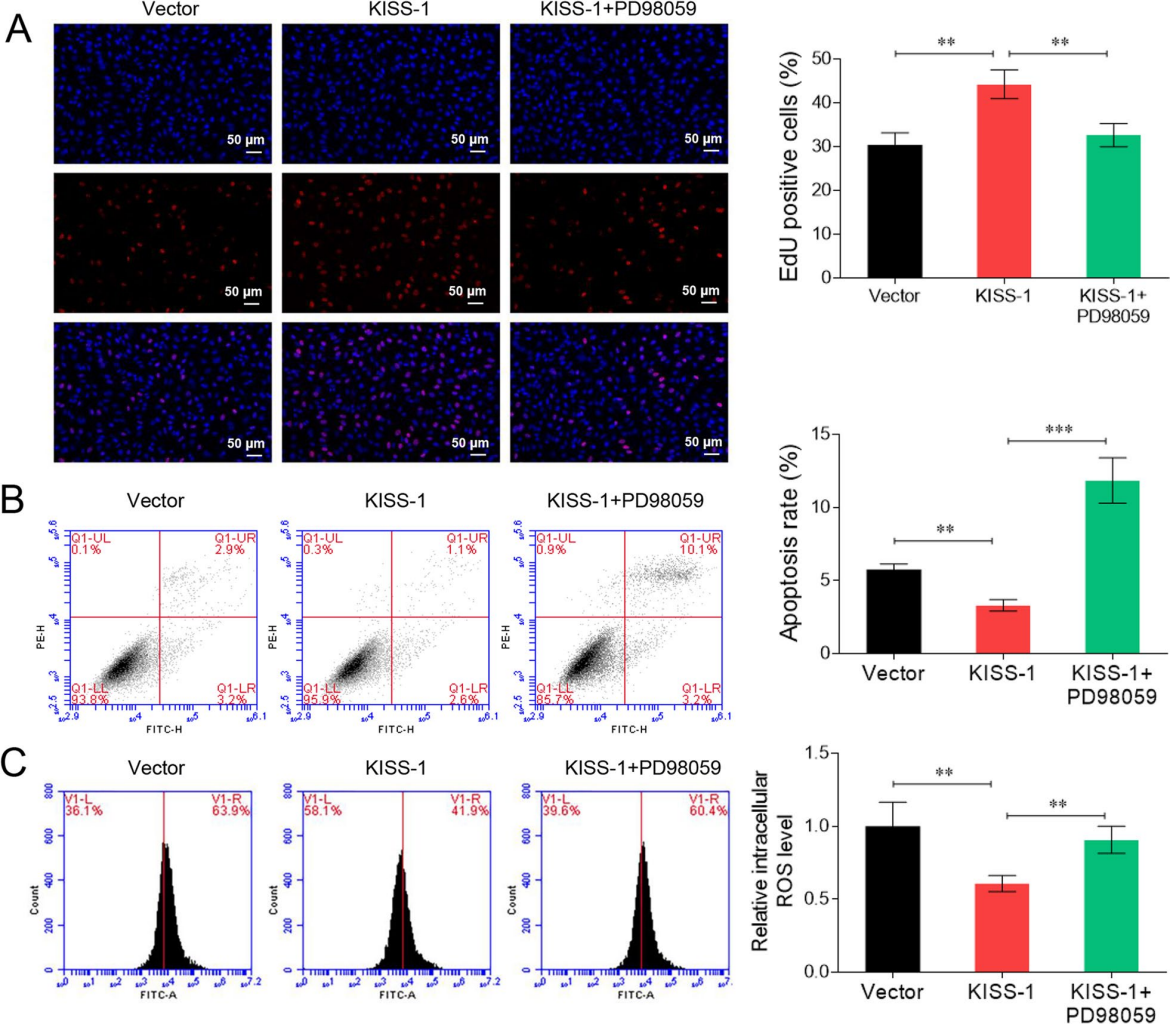
Kisspeptin promotes KGN cell survival via activation of PI3K/AKT/ERK signalling. KGN cells were transfected with KISS-1-overexpression vector and treated with an ERK1/2 antagonist PD98059. The cell viability (**A**), apoptosis (**B**), and ROS generation (**C**) were examined. **P < 0.01, ***P < 0.001

We confirmed the kisspeptin/PI3K axis-mediated ovarian granulosa cell behaviour using the ERK1/2 antagonist PD98059. As shown in Fig. 6A, B, treatment with PD98059 decreased kisspeptin-enhanced cell proliferation and eliminated the anti-apoptotic effects of kisspeptin (*P* < 0.05). Moreover, the decrease in ROS levels induced by kisspeptin overexpression was reversed by treating the cells with PD98059 (*P* < 0.05, Fig. 6C). These data demonstrate that kisspeptin modulates ovarian granulosa cell proliferation, apoptosis, and oxidative stress via the ERK1/2 signalling cascade.

## Discussion

Although PCOS is a common gynaecological disease in women [21] the mechanisms involved in the aetiology and pathology of PCOS remain elusive. Altered gene expression may play an essential role in PCOS initiation and progression [22]. The DHEA-stimulated rat model presents ovarian, endocrine, and metabolic features that are similar to those of human PCOS, such as polycystic ovaries, excessive androgen, and glucose intolerance [23]; therefore, it is widely used in PCOS studies [24–26]. Here, we examined the expression of kisspeptin in the PCOS rat model and observed significantly decreased kisspeptin RNA and protein levels in ovarian granular cells of DHEA-stimulated PCOS rats compared with rats from the control group. Additionally, the beneficial effects of kisspeptin on promoting KGN cell proliferation, cell cycle progression, and inhibition of cell apoptosis and oxidative stress were observed in vitro. These results suggest a potential protective role of kisspeptin in PCOS, which is consistent with its important role in controlling male hormone levels and maintaining the normal function of the reproductive system [27].

Kisspeptin is an essential regulator of reproductive function and its expression is closely associated with precocious puberty [28]. Kisspeptin expression is also considered a marker for PCOS diagnosis. A meta-analysis comprising 660 PCOS patients and 600 controls demonstrated a higher blood concentration of kisspeptin in patients with PCOS than in controls, and kisspeptin levels differed between non-overweight and obese patients [29]. However, kisspeptin expression was lower in the mural granulosa and cumulus cells of women with PCOS than in healthy controls [30]. Similarly, hypothalamic kisspeptin expression was reduced in a dihydrotestosterone-induced rat model of PCOS [31]. Hypothalamic downregulation of kisspeptin expression was also confirmed in testosterone- and estradiol-induced rat models [32]. In this study, we observed lower kisspeptin expression in the ovarian granular cells of DHEA-stimulated PCOS rats. Therefore, different areas of the body or animal models of PCOS with distinct metabolic phenotypes may result in varying kisspeptin expression.

Previous studies have shown that the apoptosis and dysfunction of ovarian granulosa cells is closely correlated with PCOS [33, 34]. Improving the survival of ovarian granulosa cells is important for PCOS treatment. Lower kisspeptin expression in human granulosa cells isolated from patients with PCOS contributes to abnormal development and ovulation of the ovary during PCOS [30]. The addition of kisspeptin to porcine ovarian cells results in cell proliferation and inhibition of apoptosis [35]. We observed that kisspeptin overexpression promoted proliferation while inhibiting apoptosis and oxidative stress in the ovarian granulosa cell line KGN, whereas kisspeptin knockdown led to opposite outcomes. Therefore, we speculated that kisspeptin inhibits PCOS progression by alleviating apoptosis in ovarian granulosa cells.

Oxidative stress leads to the abnormal accumulation of ROS, which is commonly caused by an imbalance between pro-oxidants and antioxidants [6]. Excessive oxidative stress in ovarian granulosa cells leads to apoptosis [36, 37]. Several studies have reported the accumulation of oxidative circulating markers in patients with PCOS and have been considered as potential inducers of PCOS pathogenesis [3, 6]. We showed that kisspeptin overexpression alleviated the accumulation of ROS and MDA (the product of lipid peroxidation) and enhanced the levels of the antioxidants GSH, SOD, and CAT in KGN cells, indicating that Kisspeptin protects ovarian granulosa cells from oxidative stress. Further studies are required to investigate the effects of kisspeptin on other aspects of ovarian granular cells, including inflammation, as the release of cytokines from ovarian granular cells plays an essential role in the pathogenesis of PCOS [38, 39].

Further evaluation of the molecular mechanisms suggested that kisspeptin activated the PI3K/AKT and ERK signalling pathways, and inhibition of ERK by an antagonist suppressed the pro-proliferative and antioxidative functions of kisspeptin. Studies have demonstrated that PI3K/AKT signalling modulates downstream pro- and anti-apoptotic genes, including Bax, caspase-3, and Bcl-2, which play critical roles in modulating the proliferation and apoptosis of ovarian granulosa cells [40–43]. However, we demonstrated a link between kisspeptin and PI3K/AKT/ERK signalling pathway in ovarian granulosa cells for the first time.

Inositol is an important component of structural lipids, namely phosphatidyl-inositol (PI) and its phosphates, including phosphatidyl-inositol phosphate (PIP) lipids. Activation of PI3K depends on the binding of the p110 and p85 subunits, which convert PIP2 to PIP3. Inositols are known to exhibit a various critical activities in several human diseases by modulating different signalling pathways, including PI3K/AKT [44]. Inositols reduce PI3K levels, thus counteracting the activation of the PKC/RAS/ERK pathway downstream of PI3K activation [45]. In insulin resistance-related human diseases, such as PCOS, defective AKT phosphorylation strongly depends on deregulated inositols availability [46]. Currently, myo-inositol is considered effective in treating infertility and menstrual irregularities in patients with PCOS [44, 47]. The correlation between inositols, PI3K/AKT, and kisspeptin in PCOS should be highlighted to further understand the biochemical network shared by PCOS metabolism.

## Conclusion

Our study indicated that kisspeptin expression was significantly decreased in the ovarian granulosa cells of PCOS rats. Overexpression of Kisspeptin improved proliferation, alleviated apoptosis of ovarian granulosa cells, and decreased ROS generation and oxidative stress through activation of the PI3K/AKT and ERK signalling pathway.

## Data Availability

The datasets generated and/or analysed during the current study are not publicly available due the article is not published but are available from the corresponding author on reasonable request.

## Abbreviations

CAT: Catalase
CCK-8: Cell counting kit-8
DHEA: Dehydroepiandrosterone
EdU: 5-Ethynyl-2′-deoxyuridine
HE: Hematoxylin and eosin
GSH: Glutathione
MDA: Malondialdehyde
ROS: Reactive oxygen species
PCOS: Polycystic ovary syndrome
SOD: Superoxide dismutase
